# FEASIBILITY OF HIGH-INTENSITY FUNCTIONAL STRENGTH TRAINING AT HOME FOR POSTINJURY OLDER ADULTS

**DOI:** 10.1093/geroni/igad104.2457

**Published:** 2023-12-21

**Authors:** Ashley Morgan, Jennifer Heisz, Ada Tang, Lehana Thabane, Julie Richardson

**Affiliations:** McMaster University, Hamilton, Ontario, Canada; McMaster University, Hamilton, Ontario, Canada; McMaster University, Hamilton, Ontario, Canada; McMaster University, Hamilton, Ontario, Canada; McMaster University, Hamilton, Ontario, Canada

## Abstract

Older adults who experience a slip, trip, or fall may experience preclinical mobility limitation (PCML), where individuals report modifications but not difficulty in mobility tasks and are at increased risk for future functional decline. Functional decline may be prevented with exercise. The purpose of this pilot trial was to determine the feasibility (adherence, recruitment, retention, safety) and preliminary outcomes (physical functioning, cognitive functioning, enjoyment) of home-based 12-week high-intensity functional strength training (HIFST) for community-dwelling older adults (≥ 55 years) with PCML who have had an injury from a slip, trip, or fall in the previous year. The trial is currently underway (target completion spring 2023). Participants are stratified by sex and randomized (1:1) to HIFST or a stretching program, both delivered by a physiotherapist via videoconferencing. Feasibility will be assessed based on predetermined criteria and reported using descriptive statistics. Preliminary effects will be reported as between group differences. To date, 20 participants (11 HIFST, 9 stretch) have enrolled (target n=24). Six participants have completed the HIFST intervention, and 2 have withdrawn before completion (reasons: mental health crisis and acute knee pain episode) with a total adherence rate of 85.8% of sessions completed (97.7% for the 6 participants who did not withdraw) which exceeds our threshold for feasibility (≥ 70%). No serious intervention-related adverse events have been reported. The results of this pilot will provide essential information for future research regarding the process, resources, and potential effects of home-based HIFST in a PCML post-injury older adult population.

